# Supplementary material for: Causal Relationship between Aging and Anorexia Nervosa: A White-Matter-Microstructure-Mediated Mendelian Randomization Analysis
Source: Biomedicines. 2024 Aug 16;12(8):1874. doi: 10.3390/biomedicines12081874 (PMC11351342; doi:10.3390/biomedicines12081874)
Supplement: Supplementary file 1 [file biomedicines-12-01874-s001.zip › Supplementary Figure/Figure S7/Single SNP plot of MR analysis for ubm-b-1768 to AN.pdf]

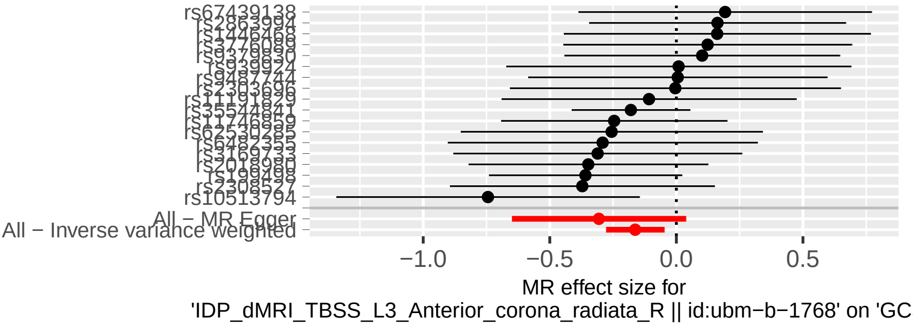
rs67439138
rs2863994
rs1446468
rs3776089
rs9379830
rs939924
rs9487744
rs2303696
rs111911829
rs35544841
rs117468859
rs62530285
rs6482355
rs3169733
rs2018980
rs199498
rs2308527
rs10513794
All − MR Egger
All − Inverse variance weighted
−1.0
−0.5
0.0
0.5
MR effect size for
'IDP_dMRI_TBSS_L3_Anterior_corona_radiata_R || id:ubm−b−1768' on 'GC
